# The Down syndrome brain in the presence and absence of fibrillar β-amyloidosis

**DOI:** 10.1016/j.neurobiolaging.2017.01.009

**Published:** 2017-05

**Authors:** Tiina Annus, Liam R. Wilson, Julio Acosta-Cabronero, Arturo Cardenas-Blanco, Young T. Hong, Tim D. Fryer, Jonathan P. Coles, David K. Menon, Shahid H. Zaman, Anthony J. Holland, Peter J. Nestor

**Affiliations:** a Cambridge Intellectual and Developmental Disabilities Research Group, Department of Psychiatry, University of Cambridge, Douglas House, Cambridge, UK; b German Center for Neurodegenerative Diseases (DZNE), Magdeburg, Germany; c Wellcome Trust Centre for Neuroimaging, Institute of Neurology, University College London, London, UK; d Wolfson Brain Imaging Centre, Department of Clinical Neurosciences, University of Cambridge, Cambridge, UK; e Division of Anaesthesia, Department of Medicine, University of Cambridge, Cambridge, UK; f Cambridgeshire and Peterborough NHS Foundation Trust, Elizabeth House, Fulbourn Hospital, Fulbourn, Cambridge, UK

**Keywords:** Alzheimer's disease, Amyloid, Cortical thickness, Down syndrome, Gray matter volume

## Abstract

People with Down syndrome (DS) have a neurodevelopmentally distinct brain and invariably developed amyloid neuropathology by age 50. This cross-sectional study aimed to provide a detailed account of DS brain morphology and the changes occuring with amyloid neuropathology. Forty-six adults with DS underwent structural and amyloid imaging—the latter using Pittsburgh compound B (PIB) to stratify the cohort into PIB-positive (n = 19) and PIB-negative (n = 27). Age-matched controls (n = 30) underwent structural imaging. Group differences in deep gray matter volumetry and cortical thickness were studied. PIB-negative people with DS have neurodevelopmentally atypical brain, characterized by disproportionately thicker frontal and occipitoparietal cortex and thinner motor cortex and temporal pole with larger putamina and smaller hippocampi than controls. In the presence of amyloid neuropathology, the DS brains demonstrated a strikingly similar pattern of posterior dominant cortical thinning and subcortical atrophy in the hippocampus, thalamus, and striatum, to that observed in non-DS Alzheimer's disease. Care must be taken to avoid underestimating amyloid-associated morphologic changes in DS due to disproportionate size of some subcortical structures and thickness of the cortex.

## 1 Introduction

People with Down syndrome (DS) are known to have developmentally altered brain structure caused by trisomy of chromosome 21. Children with DS present with delayed maturation of the central nervous system, which has been linked to prenatal arrest of neurogenesis and synaptogenesis (Schmidt-Sidor et al., 1990, Wisniewski, 1990). Postmortem studies in adults with DS have found several brain abnormalities, including reduced gross brain weight, a lower number and depth of cerebral sulci, enlarged ventricles and hypoplasia of several brain structures such as the brainstem, cerebellum, frontal and temporal lobes. In contrast, subcortical structures are shown to be relatively preserved (De La Monte and Hedley-Whyte, 1990, Delabar et al., 2006, Dierssen, 2012, Dierssen and Ramakers, 2006, Lott and Dierssen, 2010). Imaging studies in adults with DS have corroborated the postmortem findings by showing widespread cerebral hypoplasia and ventricular enlargement in comparison to typically developing individuals (Supplementary Table 1, Aylward et al., 1997a, Aylward et al., 1997b Aylward et al., 1999, Beacher et al., 2009, Beacher et al., 2010, Frangou et al., 1997, Kesslak et al., 1994, Koran et al., 2014, Krasuski et al., 2002, Pearlson et al., 1998, Pinter et al., 2001, Prasher et al., 2003, Raz et al., 1995, Roth et al., 1996, Teipel et al., 2003, Teipel et al., 2004, White et al., 2003). The vast majority of neuroimaging studies, however, are based on region-of-interest volumetry, which is only able to detect volumetric changes in predetermined regions. There is a need to study the structural morphology of the whole DS brain in a more unbiased way.

In addition to the developmental abnormalities, people with DS are at high risk for early-onset Alzheimer's disease (AD) and have been found to deposit β-amyloid plaques from about 40 years of age (Annus et al., 2016, Mann et al., 1984, Mann et al., 1990). Similar to sporadic AD, significant amyloid binding with positron emission tomography (PET) is found before any signs of cognitive or functional decline in DS (Annus et al., 2016, Handen et al., 2012, Hartley et al., 2014, Landt et al., 2011). Yet, previous structural imaging studies (see Supplementary Table 1) have aimed to characterize the developmental alterations of the adult DS brain by studying non-demented individuals; such studies are, therefore, potentially confounded through the aggregation of amyloid-positive and -negative participants. For instance, 4 previous neuroimaging studies (Aylward et al., 1999, Beacher et al., 2009, Pearlson et al., 1998, Prasher et al., 2003) aimed to characterize cerebral atrophy associated with AD in DS by comparing cognitively stable individuals to those with a clinical diagnosis of dementia. It is highly likely, however, that a sizable proportion of cognitively stable individuals already had amyloidosis, and it is known from the general population (Desikan et al., 2010, Dickerson and Wolk, 2011, Dickerson et al., 2009) that abnormalities in cortical structure occur at presymptomatic stages of AD. As such, the present cross-sectional study aimed to characterize the morphology of the adult DS brain in the absence of amyloid deposits and to describe the changes seen in the presence of fibrillar β-amyloid neuropathology.

## 2 Materials and methods

### 2.1 Study design and participants

Forty-six adults with DS and 30 typically developing participants (controls) took part in the present study. The DS cohort is the same as that reported in a previous amyloid PET study (Annus et al., 2016) with the exception of 2 amyloid-negative and 1 amyloid-positive participant, whose magnetic resonance imaging (MRI) scans were of inferior quality (motion artifact evident on visual inspection) and hence unsuitable for reliable morphometric analysis. Adults with DS were identified via clinical and social services for people with intellectual disabilities in England and Scotland and via the DS Association (UK), whereas volunteers with typical neurodevelopment were recruited from the local community via advertisement. Control participants were screened to exclude neurological and major psychiatric illness and developmental disorders. All study participants were screened for contraindications to MRI. Written consent was obtained from typically developing controls and all adults with DS with capacity to consent. Verbal assent was obtained from participants with DS lacking capacity, and a written assent was provided by an appointed consultee, in accordance with the UK Mental Capacity Act (2005). Ethics and research and development approvals were granted by the National Research Ethics Committee of East of England–Norfolk and Cambridgeshire and Peterborough NHS Foundation Trust, respectively.

### 2.2 Clinical assessments

All participants with DS had previously received a clinical diagnosis of DS based on the characteristic phenotype with full trisomy 21 confirmed in 33 DS participants by karyotyping. All participants with DS were assessed for dementia using the Cambridge Examination for Mental Disorders in Older people with DS and Others with Intellectual Disabilities informant interview as described previously (Annus et al., 2016) and allocated into categories of “stable cognition”, “cognitive decline”, and “dementia”. Dementia was diagnosed in accordance with the International Classification of Diseases-10 criteria and diagnosis of “cognitive decline” was given to participants with evidence of functional decline in one or more cognitive domains, whereas insufficient to satisfy the full criteria for dementia. All DS participants, except 3 who had severe dementia and were untestable, were administered the cognitive function assessment–CAMCOG–part of the Cambridge Examination for Mental Disorders in Older people with DS and others with Intellectual Disabilities. With the exception of 1 demented individual who was receiving Donepezil, no participants were using antidementia medications.

### 2.3 Magnetic resonance imaging acquisition

All participants underwent an anatomical MRI scan on a Siemens Verio 3T scanner with 12-channel head coil (Siemens AG, Erlangen, Germany) using the 3D T1-weighted magnetization–prepared, rapid gradient echo pulse sequence with the following parameters: repetition time/echo time/inversion time/flip angle = 2300 ms/2.98 ms/900 ms/9°, 256 × 240 × 176 matrix dimensions and 1 × 1 × 1 mm^3^ voxel size. Receiver bandwidth and echo spacing were 240 Hz/pixel and 7.1 ms, respectively, and parallel acceleration was disabled. The imaging protocol included whole-brain, T2-weighted, half-Fourier acquisition, single-shot turbo spin echo sequence (repetition time/echo time/flip angle/turbo factor = 1500 ms/79 ms/150°/256; 0.9 × 0.7 × 4.0 mm^3^ voxel size) to assess for vascular pathology and incidental lesions. For all acquisitions, the field of view was aligned in stereotactic space, with the axial plane aligned to the anterior commissure–posterior commissure line and the sagittal plane to the interhemispheric fissure.

### 2.4 Positron emission tomography using [11C]–Pittsburgh compound B

Details of the PIB PET data acquisition and processing have been published previously (Annus et al., 2016). Briefly, PIB PET images were acquired in 3D mode on a GE Advance scanner (General Electric Medical Systems, Milwaukee, WI, USA) for 90 minutes post-PIB injection in 58 frames. Only participants with DS were assessed for amyloid. Cortical regional PIB analysis was based on Brodmann areas, whereas subcortical regions of interest were based on deep gray matter parcelations using FIRST (Patenaude et al., 2011) and included the striatum (caudate nucleus and putamen), amygdala, thalamus, and hippocampus. For each region of interest, nondisplaceable binding potential was obtained using a basis function implementation of the simplified reference tissue model (Gunn et al., 1997) with superior cerebellar gray matter as reference region. PIB-positive and PIB-negative groups were assigned on the basis of striatal nondisplaceable binding potential, which had previously revealed a bimodal distribution with clear separation of positive and negative groups (Annus et al., 2016). Of the 46 participants with DS, 19 were PIB-positive.

### 2.5 Image processing

#### 2.5.1 Cortical thickness analysis

Cortical thickness analysis was conducted using FreeSurfer (v5.3, available from https://surfer.nmr.mgh.harvard.edu). The detailed procedure for surface reconstruction and estimation of cortical thickness has been described previously (Dale et al., 1999, Fischl and Dale, 2000, Fischl et al., 1999). Image processing involved automated nonuniformity bias correction, skull stripping, segmentation of the white matter, and estimation of the gray/white matter boundary. Segmented and skull-stripped data of all participants were visually inspected for parcelation errors, and topological defects in the gray/white matter boundary were manually corrected. The gray/white boundary served as a starting point for a deformable surface algorithm to compute the gray/white and pial surfaces, from which cortical thickness is calculated as the closest distance from the gray/white matter boundary to the pial surface at each vertex on the tessellated surface. Thickness measurements were mapped on the inflated surface of each participant's brain reconstruction for visualization of data across the entire cortical surface. The thickness measurements using this technique have been shown to be both valid (Rosas et al., 2002) and reliable (Dickerson et al., 2008). Each participant's brain was then morphed and aligned to a spherical common surface template using a high-resolution surface-based averaging technique that aligns cortical folding patterns by individual sulci and gyri. Before analysis, the data were smoothed with a 25 mm full-width at half-maximum Gaussian kernel, the rationale for using a large kernel size has been published previously (Diaz-de-Grenu et al., 2014). Statistical analysis was performed using the QDEC module implemented in FreeSurfer. For each hemisphere, General Linear Model was applied to estimate variations in cortical thickness at each vertex of the surface. Statistical surface maps were created using a vertexwise statistical threshold of *p* < 0.05 corrected for false discovery rate at q < 0.05. Covariate of interest analysis in FreeSurfer demonstrated no significant gender or total intracranial volume (TIV) specific regional differences in cortical thickness between PIB-negative group and neurotypical controls, resulting in the exclusion of both covariates from the statistical models (data not shown). Furthermore, β-amyloid has been shown to be a function of age in people with DS (Annus et al., 2016) and by controlling for age, the true effects of AD would be removed. A subgroup analysis was conducted in age-matched PIB-negative and PIB-positive group by including all individuals, who were in the age bracket of 39–48 years representing the time from first evidence of amyloid binding to the whole cohort being positive on PIB PET (for detailed results, see Annus et al., 2016).

#### 2.5.2 Deep gray matter volumetry

Deep brain regions of interest were defined by constructing a mask of these structures using FIRST (Patenaude et al., 2011, available from: http://fsl.fmrib.ox.ac.uk/fsl/fslwiki/FIRST). Individual structural neuroimaging data were registered by a 2-stage affine transformation to a standard Montreal Neurological Institute space with 1 mm^3^ resolution using 12 degrees of freedom, after which the registered data was fitted with “a surface mesh” modeled using a multivariate Gaussian model based on the shape and intensity information from the normalized T1-images (Patenaude et al., 2011). The segmentation was performed on all subcortical structures using the run_first_all wrapper with default settings and visually inspected for errors and misregistrations. As a result, nucleus accumbens, brainstem, and globus pallidus were excluded from the analyses due to their small size, poor segmentation, and high frequency of calcified lesions, respectively. Volumes of the structures of interest (putamen, caudate nucleus, thalamus, hippocampus, and amygdala) for each hemisphere were calculated and corrected for TIV (Jack et al., 1989). To limit the number of statistical tests, volumes of the left and right hemisphere structures were averaged for group comparisons. TIV and total brain volumes were determined by summing the relevant tissue classes using Statistical Parametric Mapping (Pengas et al., 2009). All calculated volumes were normally distributed across study groups (Shapiro-Wilk test, all *p* > 0.05), thus parametric tests in SPSS Statistics 22.0 (IBM, Corp) were implemented for statistical analyses. Between-group differences were assessed using 1-way analysis of variance, whereas independent group and correlation analyses were conducted using independent sample *t*-test (all 2-tailed) and Pearson's correlation test, respectively. The relationship between categorical variables was assessed using chi-squared test. A significance level of *p* < 0.05 was used for all inferences with results reported as mean and standard deviation.

## 3 Results

### 3.1 Demographics and summary measures

Participant demographics are provided in Table 1. Participants in the PIB-negative group were significantly younger than controls [t(48) = −4.311, *p* < 0.001] and the PIB-positive group [t(37) = −6.722, *p* < 0.001], whereas controls and PIB-positive group were of similar ages [t(47) = 1.491, *p* = 0.143]. The control group had significantly larger TIV than PIB-negative [t(55) = 7.370, *p* < 0.001] and PIB-positive [t(47) = 5.535, *p* < 0.001] individuals with DS, whereas there was no difference in TIV between the 2 DS groups [t(44) = 0.735, *p* = 0.466]. The PIB-positive group had significantly lower TIV-corrected total brain volume than PIB-negative group [t(44) = −5.304, *p* < 0.001], as well as controls [t(47) = −4.444, *p* < 0.001]. PIB-negative and control groups had similar TIV-corrected total brain volume [t(55) = −0.189, *p* = 0.850]. Furthermore, on average, the isocortex in the PIB-negative group was 4.3% thicker than that in controls and 4.9% thicker than that in the PIB-positive group, whereas PIB-positive and control group had similar mean global cortical thickness (0.56% lower in PIB-positive, Supplementary Fig. 1). No differences were observed in the cognitive function test CAMCOG between PIB-negative and PIB-positive groups [t(41) = −1.057, *p* = 0.297]. Note, however, that 3 individuals in the PIB-positive group were untestable because of dementia—for this reason, analyses were also run excluding these 3 individuals to contrast PIB-positive and PIB-negative participants matched for CAMCOG performance. Excluding these 3 severely demented individuals did not influence the findings reported for the overall PIB-positive group (Table 1). Interestingly, even the cognitively stable participants in the PIB-positive group had significantly lower TIV-corrected total brain volume [1087.98 ± 72.82 cm^3^ vs. 1148.05 ± 31.27 cm^3^, respectively; t(29) = −3.232, *p* = 0.003] and lower mean global cortical thickness [2.58 ± 0.10 mm vs. 2.68 ± 0.10 mm, respectively; t(29) = 2.425, *p* = 0.022] than cognitively stable participants in the PIB-negative group.

### 3.2 Cortical thickness

#### 3.2.1 PIB-negative Down syndrome versus controls

**Fig. 1:**
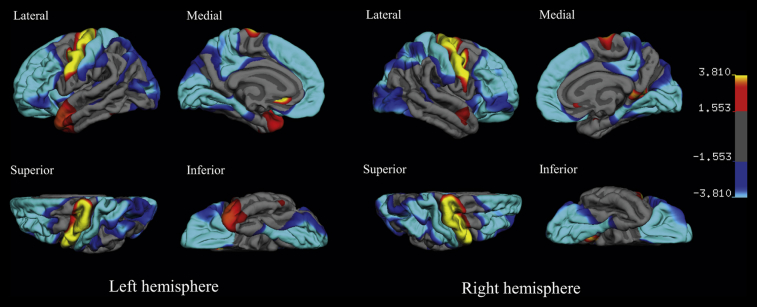
The cortical signature of the Down syndrome brain without amyloid pathology: regional variations in cortical thickness across the hemispheres in the PIB-negative group (n = 27) in comparison to control group (n = 30). The color scale on the right represents the significance of the thickness difference as −log 10 (*p*-value) with red-yellow indicating thinner cortex and blue-light blue indicating thicker cortex in the PIB-negative group relative to controls. The results are false discovery rate corrected at *p* < 0.05.

The vertexwise analysis in the PIB-negative group compared with controls demonstrated highly significant cortical thinning in the precentral gyrus; significant thinning was also found in the temporal pole with additional small areas in subgenual anterior cingulate and retrosplenial cortex (Fig. 1). Widespread cortical thickness increases relative to controls were evident in medial and lateral prefrontal cortex; parietal, precuneus, and posterior cingulate cortex; postcentral gyrus; occipital and posteroinferior temporal cortex. Controlling for age differences between PIB-negative and control group did not alter the main findings (see Supplementary Fig. 2).

#### 3.2.2 PIB-positive Down syndrome versus PIB-negative Down syndrome

**Fig. 2:**
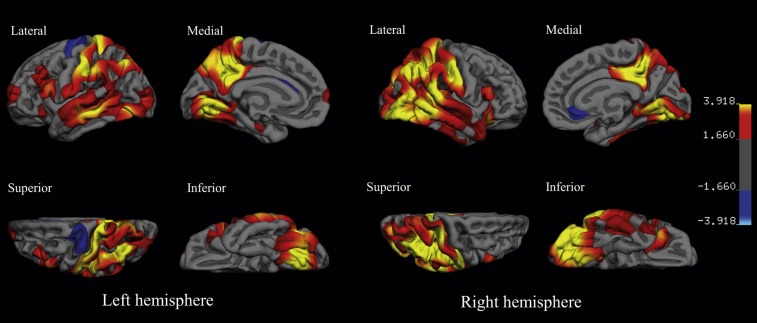
The cortical signature of the Down syndrome brain with amyloid pathology: regional variations in cortical thickness in the PIB-positive group (n = 19), when compared to PIB-negative group (n = 27). The color scale on the right represents the significance of the difference in thickness as −log 10 (*p*-value) with red-yellow indicating thinner cortex and blue-light blue indicating thicker cortex in the PIB-positive group relative to PIB-negative group. The results are false discovery rate corrected at *p* < 0.05.

Vertexwise analysis of cortical thickness in the PIB-positive compared to PIB-negative group revealed large confluent clusters of cortical thinning in parieto-temporo-occipital cortices, posterior cingulate, and precuneus cortices along with some small and scattered changes in prefrontal areas. The extent of cortical thinning was more marked in the right hemisphere. In contrast, there were some small, weakly significant areas of greater thickness in the PIB-positive group in the right subgenual cortex and left precentral gyrus (Fig. 2). Removal of the 3 PIB-positive DS individuals, who were too advanced to complete the CAMCOG, did not alter the reported findings (data not shown). Although PIB-negative group was significantly younger than PIB-positive, matching the groups for age is problematic because, by definition, transition to amyloid positivity is a function of age in people with DS (see Annus et al., 2016). Nonetheless, the age bracket from 39 to 48 years contained an overlap of PIB-positive (n = 9) and PIB-negative (n = 13) participants that were matched for age [t(20) = 1.481, *p* = 0.154]. Reducing the power by only looking at this subgroup meant that the significance of the changes diminished, however at *p* (uncorr) < 0.05, a similar atrophy pattern to that seen in the full cohort contrast was evident (Supplementary Fig. 3).

### 3.3 Deep gray matter volumetry

**Fig. 3:**
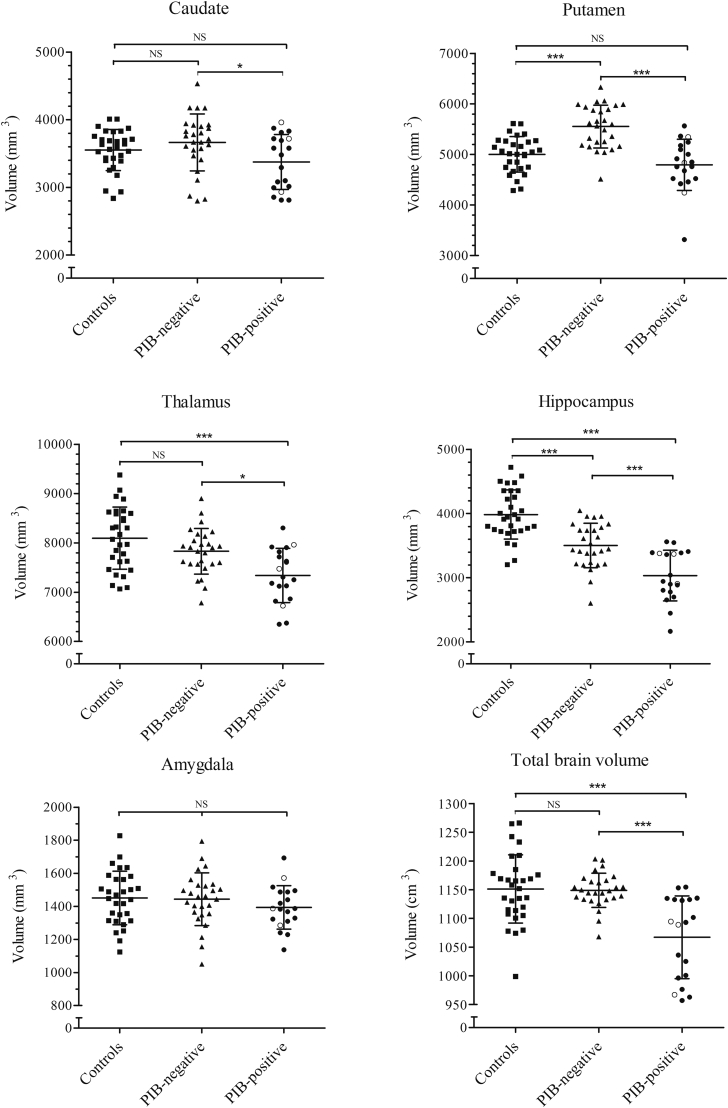
Volumes of the deep gray matter structures and total brain in the control, PIB-negative and PIB-positive groups. Unfilled circles in the PIB-positive group represent the 3 individuals who had too advanced dementia to be able to perform the CAMCOG cognitive assessment. ^∗^*p* < 0.05, ^∗∗∗^*p* < 0.001, NS, nonsignificant. All tests are 2-sample *t*-test results (2-tailed) following a 1-way analysis of variance. Abbreviation: CAMCOG, Cambridge Examination for Mental Disorders in Older people with DS and others with Intellectual Disabilities.

Compared to controls, the PIB-negative group had significantly larger TIV-corrected putamina [t(55) = 5.351, *p* < 0.001] and smaller hippocampi [t(55) = 4.951, *p* < 0.001], whereas no volumetric differences were identified in the caudate nucleus and thalamus. The results remained unaltered, when corrected for differences in age between the 2 groups (data not shown). Relative to the PIB-negative group, the PIB-positive group demonstrated significantly atrophic caudate nucleus [t(44) = 2.323, *p* < 0.05], putamen [t(44) = 5.52, *p* < 0.001], thalamus [t(44) = 3.277, *p* = 0.01], and hippocampus [t(44) = 4.258, *p* < 0.001], whereas only thalamic [t(47) = 4.289, *p* < 0.001] and hippocampal volumes [t(47) = 8.361, *p* < 0.001] were smaller when compared to controls. There were no significant differences in the TIV-corrected volumes of amygdala [F(3,75) = 0.878, *p* = 0.42] across the 3 groups (Fig. 3). Furthermore, the inclusion of 3 severely demented individuals in the PIB-positive group, who were untestable on the CAMCOG assessment, had no impact on the deep gray matter volumetry findings in the PIB-positive group (unfilled circles, Fig. 3).

## 4 Discussion

The present study demonstrated a distinct neurodevelopmental phenotype of the DS brain with evidence of a thicker cortical ribbon particularly in the frontal and occipital lobes and thinner motor cortex. Development of fibrillar β-amyloidosis in people with DS is associated with a widespread posterior cortical thinning and subcortical atrophy in a pattern highly concordant with that seen in sporadic (Dickerson et al., 2009, Fjell et al., 2014, Jack et al., 2009) and familial AD (Cash et al., 2013, Fortea et al., 2010). This study is the first of its kind to provide a comprehensive analysis of the cortical and subcortical landscape of the adult DS brain in groups clearly defined according to amyloid status.

### 4.1 The structure of the Down syndrome brain without amyloid

It is expected that the DS brain differs qualitatively from that of the typically developing population. The aim of the contrast of amyloid-negative DS to healthy controls was to map these differences and, therefore, to aid interpretation of the changes that then occur in the DS brain in the presence of amyloid. With a mean age of 46 years, it was considered highly unlikely that the control group would be contaminated with amyloid-positive participants. In line with previous work (see Supplementary Table 1), adults with DS presented with significantly lower TIV compared to neurotypical controls. The current findings indicate, however, that the DS brain is not simply a downscale model of the typically developed brain but has a distinct topography of regions that are disproportionally smaller or larger with respect to the smaller brain size. In the absence of amyloid, the brain of people with DS is characterized by atypically and disproportionally thicker cortex in the frontal, parietal, occipitotemporal, and posterior cingulate areas. Furthermore, people with DS have neurodevelopmentally thinner motor cortices and temporal poles. The present findings are in line with a recent report of increased cortical thickness in the frontal, parietal, and occipital lobes in children and young adults with DS (mean age: 15 years, range: 5–24 years; Lee et al., 2016), demonstrating that a largely thicker cortex is present already in childhood and persists into adulthood in individuals with DS. However, the authors only focused on the pattern of thicker cortex in the DS youth in comparison to typically developing youth and did not investigate the reverse contrast, thus possibly missing the pattern of cortical thinning described in the present study. Interestingly, the regions that are atypically thicker in DS are characterized by pronounced cortical thinning during brain development in adolescents without trisomy 21 (Vandekar et al., 2015). This suggests a delay in the cortical thinning in DS and that the abnormalities in the development and maturation of the cortex extend beyond early development well into adulthood.

In deep gray matter structures, the most striking differences between people with DS and neurotypical controls was in the striatum—specifically that the putamen was disproportionately enlarged in amyloid-negative adults with DS. Disproportionally greater volume of the putamen has been previously found in a morphometry study of children (Menghini et al., 2011) and in nondemented adults with DS (Aylward et al., 1997b, Beacher et al., 2010), when compared to typically developing individuals. This again points to a neurodevelopmental origin of the observed structural enlargement, in line with the findings of increased cortical thickness as a result of abnormal cortical development and maturation in DS.

The hippocampi were disproportionally smaller in PIB-negative individuals with DS compared to neurotypical controls—a finding consistent with previous in vivo volumetry reports (Kesslak et al., 1994, Krasuski et al., 2002, Menghini et al., 2011, Raz et al., 1995, Smigielska-Kuzia et al., 2011, White et al., 2003). As the present study was cross-sectional, it cannot address whether this reduction in hippocampal volume in adults with DS is developmental (hypoplasia) or acquired (atrophy). It would seem, however, to almost certainly represent hypoplasia because past studies have found decreased hippocampal volume in adults younger than 30 years (Raz et al., 1995), as well as in adolescents (Menghini et al., 2011) and children (Smigielska-Kuzia et al., 2011) with DS. Although these past studies did not include amyloid imaging, it seems reasonable to assume, based on amyloid PET (Annus et al., 2016) and pathology (Mann and Esiri, 1989) studies, that such participants would have been amyloid negative. The rest of the subcortical structures, including the thalamus and the amygdala, did not exhibit any DS specific characteristics in the absence of PIB binding and were neurodevelopmentally proportional to those of typically developing controls.

### 4.2 The PIB-positive Down syndrome brain

A significant reduction in TIV-corrected total brain volume was observed in the PIB-positive group, when compared to the PIB-negative group. In addition to marked volumetric differences, amyloid-positive individuals also presented with significantly lower mean global cortical thickness than their amyloid-negative counterparts. This reduction in both volume and cortical thickness was significant even in the cognitively stable PIB-positive participants, demonstrating that, in keeping with sporadic (Aizenstein et al., 2008, Becker et al., 2011) and familial AD (Klunk et al., 2007, Villemagne et al., 2009), volume loss and cortical thinning is measurable before any clinical signs of cognitive decline in people with DS. The neocortex of PIB-positive adults with DS exhibited a pattern of posterior dominant cortical thinning mapped to the parieto–temporo-occipital cortices laterally and to the medial posterior cingulate and precuneus cortices. This pattern of reduced thickness in DS was highly similar to that observed in sporadic and familial AD (Boxer et al., 2003, Cash et al., 2013, Du et al., 2007, Fjell et al., 2014, Gili et al., 2011). The volumes of putamen and caudate were significantly lower in PIB-positive individuals with DS compared to either the PIB-negative group or typically developing controls. This result highlights the importance of amyloid stratification in DS–the striatal volume reduction would be missed entirely if one examined volumetric changes in PIB-positive DS only in comparison to a neurotypical control group. The striatum has been receiving increasing interest in the field of AD, particularly as it has been shown to be the first site of PET amyloid binding in the familial forms of early onset AD (Klunk et al., 2007, Koivunen et al., 2008, Pievani et al., 2013, Remes et al., 2008, Villemagne et al., 2009) and recently in people with DS (Annus et al., 2016, Handen et al., 2012).

The PIB-positive group demonstrated significant reduction in the thalamic volume, when compared to amyloid negative individuals. PIB positivity was also associated with marked hippocampal volume reduction, when contrasted to PIB-negative DS group. As already noted, the TIV–corrected hippocampal volumes in PIB-negative individuals with DS were, in turn, significantly smaller than controls. Hippocampal atrophy is an expected finding in AD, although it is interesting to note that the effect size was comparable to that for putaminal atrophy (Fig. 3) in the amyloid-positive DS group. Given that many of the amyloid-positive individuals in the present series were asymptomatic, these findings suggest that atrophy becomes measurable very early after the conversion to amyloid positivity, although this hypothesis would need to be proven in a longitudinal sample.

### 4.3 Limitations

The present study is the first in characterizing the brain morphology in neuropathologically clearly defined groups of amyloid-positive and -negative adults with DS. There were, however, inevitable age differences between the PIB-negative and PIB-positive group as a result of age-dependent threshold of amyloidosis in the DS population (Annus et al., 2016). As such, it is not possible to control for the differences in age in the statistical models without removing the effects of amyloid. Although the lack of age-matched groups is a limitation of the present study, repeating the cortical thickness analysis in smaller (n = 22) but age-matched groups of 39- to 48-year-olds yielded similar results (Supplementary Fig. 3). Although the significance (expectedly) diminished, the results were consistent with the full group analysis suggesting that they were not spurious and, in turn, making it very unlikely that the whole group results were merely driven by between-group age differences. Three individuals in the PIB-negative group were diagnosed with dementia (n = 2/3) or cognitive decline (n = 1/3) albeit having no evidence of amyloidosis in PIB-PET. Diagnosing dementia in people with intellectual disabilities (including DS) is a challenge with an acknowledged risk of uncertainty, particularly exacerbated by the presence of underlying intellectual disability, frequent lack of information about individual's premorbid level of functioning and difficulties in communication and therefore heavy reliance on informant opinion (Burt et al., 1998; for discussion see Annus et al., 2016). Furthermore, changes associated with amyloid deposition in DS are described using a cross-sectional dataset. Although longitudinal studies are needed to understand better the relationship between sub/cortical atrophy and amyloidosis, the present findings have highlighted that, in the presence of confirmed amyloidosis, the pattern of cortical thinning is similar across AD regardless of etiology. Finally, it will be of interest in future studies with greater power to examine the severity of atrophy stratified not just for amyloidosis but also with respect to clinical status (i.e., stable cognition/cognitive decline/dementia); in the present cohort (see Table 1) such a stratification resulted in groups that were too small to reliably address this issue.

## 5 Conclusion

This study is the first to describe the cortical landscape of the adult DS brain in distinct neuropathologically defined groups. Adults with DS presented with significant neuroanatomical differences from typically developing controls, which were further exacerbated in those individuals with evidence of amyloid pathology on a PIB PET scan. In the absence of amyloid, people with DS had smaller brain volume but with atypically thicker cortex in the frontal and occipitoparietal cortices and thinner motor cortex and temporal pole compared to controls. The data also demonstrated that, compared to the general population, people with DS had disproportionately larger putamina and smaller hippocampi, likely a result of abnormal brain development and maturation. In the presence of amyloidosis, adults with DS demonstrated posterior dominant cortical thinning as well as atrophy in the hippocampi, thalami, and striatum in a strikingly similar pattern as observed in sporadic and familiar AD. The data further showed that people with DS can tolerate significant cortical atrophy in the presence of amyloid without deleterious effects on their cognitive function. The present study has highlighted the importance of first understanding the neuroanatomical characteristics of the DS brain before any assessments of the effects of amyloid are undertaken. Thus, care must be taken when characterizing amyloid-related structural changes in individuals with DS to avoid underestimation of the observed morphology due to disproportionate size of some deep gray matter structures and increased thickness of the cortex.

## Disclosure statement

The authors report no conflict of interest.

## Figures and Tables

**Table 1:** Participant demographics

Measure	Controls	PIB-negative	PIB-positive	PIB-positive excl. 3 non-CAMCOG participants
*n*, f/m	30, 14/16	27, 13/14	19, 8/11	16, 6/10
Age, range (y)	46.2 ± 9.7, 30–64	37.1 ± 6, 28–48	49.7 ± 6.5, 39–65	48.1 ± 5.1, 39–56
Global cortical thickness (mm)	2.57 ± 0.09	2.68 ± 0.10	2.55 ± 0.12	2.56 ± 0.12
TIV (cm^3^)	1421.71 ± 140.58	1178.71 ± 103.13	1203.40 ± 124.10	1203.03 ± 115.00
TIV-corrected TBV^a^ (cm^3^)	1151.26 ± 59.52	1148.86 ± 29.87	1067.08 ± 72.05	1070.26 ± 73.94
Stable cogn./cogn. decline/dement.	N/A	24/1/2	7/5/7	7/5/4
CAMCOG score (max 109), range	N/A	76, 38–102	-	74, 17–95
ApoE^b^				
ε2/ε3	N/A	4	3	2
ε3/ε3	N/A	15	6	6
ε3/ε4	N/A	4	6	6
ε2/ε4	N/A	1	1	1

Data is shown as mean ± standard deviation and range, if applicable.

Key: ApoE, apolipoprotein E allele; Cogn. decline, cognitive decline; Dement., dementia; f/m, females/males ratio; N/A, not applicable; Stable cogn., stable cognition; TBV, total brain volume; TIV, total intracranial volume.

a Jack et al. (1989).

b Three individuals in the PIB-negative group and 3 in the PIB-positive group did not undergo ApoE genotyping.

## Appendix A Supplementary data

Supplementary Table 1 and Supplementary Figs. 1–3

Supplementary References

## References

[bib1] Aizenstein H.J., Nebes R.D., Saxton J.A., Price J.C., Mathis C.A., Tsopelas N.D., Ziolko S.K., James J.A., Snitz B.E., Houck P.R., Bi W., Cohen A.D., Lopresti B.J., DeKosky S.T., Halligan E.M., Klunk W.E. (2008). Frequent amyloid deposition without significant cognitive impairment among the elderly. Arch. Neurol..

[bib2] Annus T., Wilson L.R., Hong Y.T., Acosta–Cabronero J., Fryer T.D., Cardenas–Blanco A., Smith R., Boros I., Coles J.P., Aigbirhio F.I., Menon D.K., Zaman S.H., Nestor P.J., Holland A.J. (2016). The pattern of amyloid accumulation in the brains of adults with Down syndrome. Alzheimers Dement.

[bib3] Aylward E.H., Habbak R., Warren A.C., Pulsider M.B., Barta P.E., Jerram M., Pearlson G.D. (1997). Cerebellar volume in adults with Down syndrome. Arch. Neurol..

[bib4] Aylward E.H., Li Q.A., Habbak R., Warren A., Pulsifer M.B., Barta P.E., Jerram M., Pearlson G.D. (1997). Basal ganglia volume in adults with Down syndrome. Psychiatry Res. Neuroimaging.

[bib5] Aylward E.H., Li Q.A., Honeycutt N.A., Warren A.C., Pulsifer M.B., Barta P.E., Chan M.D., Smith P.D., Jerram M., Pearlson G.D. (1999). MRI volumes of the hippocampus and amygdala in adults with Down's syndrome with and without dementia. Am. J. Psychiatry.

[bib6] Beacher F., Daly E., Simmons A., Prasher V., Morris R., Robinson C., Lovestone S., Murphy K., Murphy D.G.M. (2009). Alzheimer's disease and Down's syndrome: an in vivo MRI study. Psychol. Med..

[bib7] Beacher F., Daly E., Simmons A., Prasher V., Morris R., Robinson C., Lovestone S., Murphy K., Murphy D.G.M. (2010). Brain anatomy and ageing in non-demented adults with Down's syndrome: an in vivo MRI study. Psychol. Med..

[bib8] Becker J.A., Hedden T., Carmasin J., Maye J., Rentz D.M., Putcha D., Fischl B., Greve D.N., Marshall G.A., Salloway S., Marks D., Buckner R.L., Sperling R.A., Johnson K.A. (2011). Amyloid-beta associated cortical thinning in clinically normal elderly. Ann. Neurol..

[bib9] Boxer A., Rankin K., Miller B., Schuff N., Weiner M., Gorno-Tempini M. (2003). Cinguloparietal atrophy distinguishes Alzheimer disease from semantic dementia. Arch. Neurol..

[bib10] Burt D.B., Loveland K.A., Primeaux-Hart S., Chen Y.-W., Phillips N.B., Cleveland L.A., Lewis K.R., Lesser J., Cummings E. (1998). Dementia in adults with Down syndrome: diagnostic challenges. Am. J. Ment. Retard..

[bib11] Cash D.M., Ridgway G.R., Liang Y.Y., Ryan N.S., Kinnunen K.M., Yeatman T., Malone I.B., Benzinger T.L.S., Jack C.R., Thompson P.M., Ghetti B.F., Saykin A.J., Masters C.L., Ringman J.M., Salloway S.P., Schofield P.R., Sperling R.A., Cairns N.J., Marcus D.S., Xiong C., Bateman R.J., Morris J.C., Rossor M.N., Ourselin S., Fox N.C., DIAN (2013). The pattern of atrophy in familial Alzheimer disease volumetric MRI results from the DIAN study. Neurology.

[bib12] Dale A.M., Fischl B., Sereno M.I. (1999). Cortical surface-based analysis: I. Segmentation and surface reconstruction. Neuroimage.

[bib13] De La Monte S.M., Hedley-Whyte E.T. (1990). Small cerebral hemispheres in adults with Down's syndrome: contributions of developmental arrest and lesions of Alzheimer's disease. J. Neuropathol. Exp. Neurol..

[bib14] Delabar J.M., Aflalo-Rattenbac R., Creau N. (2006). Developmental defects in trisomy 21 and mouse models. Scientific World J..

[bib15] Desikan R.S., Sabuncu M.R., Schmansky N.J., Reuter M., Cabral H.J., Hess C.P., Weiner M.W., Biffi A., Anderson C.D., Rosand J., Salat D.H., Kemper T.L., Dale A.M., Sperling R.A., Fischl B. (2010). Selective disruption of the cerebral neocortex in Alzheimer's disease. PLoS One.

[bib16] Diaz-de-Grenu L.Z., Acosta-Cabronero J., Chong Y.F., Pereira J.M., Sajjadi S.A., Williams G.B., Nestor P.J. (2014). A brief history of voxel-based grey matter analysis in Alzheimer's disease. J. Alzheimers Dis..

[bib17] Dickerson B.C., Bakkour A., Salat D.H., Feczko E., Pacheco J., Greve D.N., Grodstein F., Wright C.I., Blacker D., Rosas H.D., Sperling R.A., Atri A., Growdon J.H., Hyman B.T., Morris J.C., Fischl B., Buckner R.L. (2009). The cortical signature of Alzheimer's disease: regionally specific cortical thinning relates to symptom severity in very mild to mild AD dementia and is detectable in asymptomatic amyloid-positive individuals. Cereb. Cortex.

[bib18] Dickerson B.C., Fenstermacher E., Salat D.H., Wolk D.A., Maguire R.P., Desikan R., Pacheco J., Quinn B.T., Van der Kouwe A., Greve D.N., Blacker D., Albert M.S., Killiany R.J., Fischl B. (2008). Detection of cortical thickness correlates of cognitive performance: reliability across MRI scan sessions, scanners, and field strengths. Neuroimage.

[bib19] Dickerson B.C., Wolk D.A. (2011). Dysexecutive versus amnesic phenotypes of very mild Alzheimer's disease are associated with distinct clinical, genetic and cortical thinning characteristics. J. Neurol. Neurosurg. Psychiatry.

[bib20] Dierssen M. (2012). Down syndrome: the brain in trisomic mode. Nat. Rev. Neurosci..

[bib21] Dierssen M., Ramakers G.J.A. (2006). Dendritic pathology in mental retardation: from molecular genetics to neurobiology. Genes Brain Behav..

[bib22] Du A.-T., Schuff N., Kramer J.H., Rosen H.J., Gorno-Tempini M.L., Rankin K., Miller B.L., Weiner M.W. (2007). Different regional patterns of cortical thinning in Alzheimer's disease and frontotemporal dementia. Brain.

[bib23] Fischl B., Dale A.M. (2000). Measuring the thickness of the human cerebral cortex from magnetic resonance images. PNAS.

[bib24] Fischl B., Sereno M.I., Dale A.M. (1999). Cortical surface-based analysis: II: inflation, flattening, and a surface-based coordinate system. Neuroimage.

[bib25] Fjell A.M., Westlye L.T., Grydeland H., Amlien I., Espeseth T., Reinvang I., Raz N., Dale A.M., Walhovd K.B., ADNI (2014). Accelerating cortical thinning: unique to dementia or universal in aging?. Cereb. Cortex.

[bib26] Fortea J., Sala-Llonch R., Bartres-Faz D., Bosch B., Llado A., Bargallo N., Molinuevo J.L., Sanchez-Valle R. (2010). Increased cortical thickness and caudate volume precede atrophy in PSEN1 mutation carriers. J. Alzheimers Dis..

[bib27] Frangou S., Aylward E., Warren A., Sharma T., Barta P., Pearlson G. (1997). Small planum temporale volume in Down's syndrome: a volumetric MRI study. Am. J. Psychiatry.

[bib28] Gili T., Cercignani M., Serra L., Perri R., Giove F., Maraviglia B. (2011). Regional brain atrophy and functional disconnection across Alzheimer's disease evolution. J. Neurol. Neurosurg. Psychiatry.

[bib29] Gunn R.N., Lammertsma A.A., Hume S.P., Cunningham V.J. (1997). Parametric imaging of ligand-receptor binding in PET using a simplified reference region model. Neuroimage.

[bib30] Handen B.L., Cohen A.D., Channamalappa U., Bulova P., Cannon S.A., Cohen W.I., Mathis C.A., Price J.C., Klunk W.E. (2012). Imaging brain amyloid in nondemented young adults with Down syndrome using Pittsburgh compound B. Alzheimers Dement.

[bib31] Hartley S.L., Handen B.L., Devenny D.A., Hardison R., Mihaila I., Price J.C., Cohen A.D., Klunk W.E., Mailick M.R., Johnson S.C., Christian B.T. (2014). Cognitive functioning in relation to brain amyloid-beta in healthy adults with Down syndrome. Brain.

[bib32] Jack C.R., Lowe V.J., Weigand S.D., Wiste H.J., Senjem M.L., Knopman D.S., Shiung M.M., Gunter J.L., Boeve B.F., Kemp B.J., Weiner M., Petersen R.C. (2009). Serial PIB and MRI in normal, mild cognitive impairment and Alzheimer's disease: implications for sequence of pathological events in Alzheimer's disease. Brain.

[bib33] Jack C.R., Twomey C.K., Zinsmeister A.R., Sharbrough F.W., Petersen R.C., Cascino G.D. (1989). Anterior temporal lobes and hippocampal formations: normative volumetric measurements from MR images in young adults. Radiology.

[bib34] Kesslak J.P., Nagata S.F., Lott I., Nalcioglu O. (1994). Magnetic resonance imaging analysis of age-related changes in the brains of individuals with Down's syndrome. Neurology.

[bib35] Klunk W.E., Price J.C., Mathis C.A., Tsopelas N.D., Lopresti B.J., Ziolko S.K., Bi W., Hoge J.A., Cohen A.D., Ikonomovic M.D., Saxton J.A., Snitz B.E., Pollen D.A., Moonis M., Lippa C.F., Swearer J.M., Johnson K.A., Rentz D.M., Fischman A.J., Aizenstein H.J., DeKosky S.T. (2007). Amyloid deposition begins in the striatum of presenilin-1 mutation carriers from two unrelated pedigrees. J. Neurosci..

[bib36] Koivunen J., Verkkoniemi A., Aalto S., Paetau A., Ahonen J.-P., Viitanen M., Någren K., Rokka J., Haaparanta M., Kalimo H., Rinne J.O. (2008). PET amyloid ligand [11C]PIB uptake shows predominantly striatal increase in variant Alzheimer's disease. Brain.

[bib37] Koran M.E., Hohman T.J., Edwards C.M., Vega J.N., Pryweller J.R., Slosky L.E., Crockett G., Villa de Rey L., Meda S.A., Dankner N., Avery S.N., Blackford J.U., Dykens E.M., Thornton-Wells T.A. (2014). Differences in age-related effects on brain volume in Down syndrome as compared to Williams syndrome and typical development. J. Neurodev Disord..

[bib38] Krasuski J.S., Alexander G.E., Horwitz B., Rapoport S.I., Schapiro M.B. (2002). Relation of medial temporal lobe volumes to age and memory function in nondemented adults with Down's syndrome: implications for the prodromal phase of Alzheimer's disease. Am. J. Psychiatry.

[bib39] Landt J., D'abrera J.C., Holland A.J., Aigbirhio F.I., Fryer T.D., Canales R., Hong Y.T., Menon D.K., Baron J.C., Zaman S.H. (2011). Using positron emission tomography and carbon 11-labeled Pittsburgh Compound B to image brain fibrillar beta-amyloid in adults with Down syndrome: safety, acceptability, and feasibility. Arch. Neurol..

[bib40] Lee N.R., Adeyemi E.I., Lin A., Clasen L.S., Lalonde F.M., Condon E., Driver D.I., Shaw P., Gogtay N., Raznahan A., Giedd J.N. (2016). Dissociations in cortical morphometry in youth with Down syndrome: evidence for reduced surface area but increased thickness. Cereb. Cortex.

[bib41] Lott I.T., Dierssen M. (2010). Cognitive deficits and associated neurological complications in individuals with Down's syndrome. Lancet Neurol..

[bib42] Mann D.M.A., Esiri M.M. (1989). The pattern of acquisition of plaques and tangles in the brains of patients under 50 years of age with Down's syndrome. J. Neurol. Sci..

[bib43] Mann D.M.A., Jones D., Prinja D., Purkiss M.S. (1990). The prevalence of amyloid (A4) protein deposits within the cerebral and cerebellar cortex in Down's syndrome and Alzheimer's disease. Acta Neuropathol..

[bib44] Mann D.M.A., Yates P.O., Marcyniuk B. (1984). Alzheimer's presenile dementia, senile dementia of Alzheimer's type and Down's syndrome in middle age form an age related continuum of pathological changes. Neuropathol. Appl. Neurobiol..

[bib45] Menghini D., Costanzo F., Vicari S. (2011). Relationship between brain and cognitive processes in Down syndrome. Behav. Genet..

[bib47] Patenaude B., Smith S.M., Kennedy D.N., Jenkinson M. (2011). A Bayesian model of shape and appearance for subcortical brain segmentation. Neuroimage.

[bib48] Pearlson G.D., Breiter S.N., Aylward E.H., Warren A.C., Grygorcewicz M., Frangou S., Barta P.E., Pulsifer M.B. (1998). MRI brain changes in subjects with Down syndrome with and without dementia. Dev. Med. Child Neurol..

[bib49] Pengas G., Pereira J.M.S., Williams G.B., Nestor P.J. (2009). Comparative reliability of total intracranial volume estimation methods and the influence of atrophy in a longitudinal semantic dementia cohort. J. Neuroimaging.

[bib50] Pievani M., Bocchetta M., Boccardi M., Cavedo E., Bonetti M., Thompson P.M., Frisoni G.B. (2013). Striatal morphology in early-onset and late-onset Alzheimer's disease: a preliminary study. Neurobiol. Aging.

[bib51] Pinter J.D., Eliez S., Schmitt J.E., Capone G.T., Reiss A.L. (2001). Neuroanatomy of Down's syndrome: a high-resolution MRI study. Am. J. Psychiatry.

[bib52] Prasher V., Cumella S., Natarajan K., Rolfe E., Shah S., Haque M.S. (2003). Magnetic resonance imaging, Down's syndrome and Alzheimer's disease: research and clinical implications. JIDR.

[bib53] Raz N., Torres I.J., Briggs S.D., Spencer W.D., Thornton A.E., Loken W.J., Gunning F.M., McQuain J.D., Driesen N.R., Acker J.D. (1995). Selective neuroanatomic abnormalities in Down's syndrome and their cognitive correlates: evidence from MRI morphometry. Neurology.

[bib54] Remes A.M., Laru L., Tuominen H., Aalto S., Kemppainen N., Mononen H., Nagren K., Parkkola R., Rinne J.O. (2008). Carbon 11-labeled pittsburgh compound B positron emission tomographic amyloid imaging in patients with APP locus duplication. Arch. Neurol..

[bib55] Rosas H.D., Liu A.K., Hersch S., Glessner M., Ferrante R.J., Salat D.H., van der Kouwe A., Jenkins B.G., Dale A.M., Fischl B. (2002). Regional and progressive thinning of the cortical ribbon in Huntington's disease. Neurology.

[bib56] Roth G.M., Sun B., Greensite F.S., Lott I.T., Dietrich R.B. (1996). Premature aging in persons with Down syndrome: MR findings. Am. J. Neuroradiol.

[bib57] Schmidt-Sidor B., Wisniewski K.E., Shepard T.H., Sersen E.A. (1990). Brain growth in Down syndrome subjects 15 to 22 weeks of gestational age and birth to 60 months. Clin. Neuropathol..

[bib58] Smigielska-Kuzia J., Bockowski L., Sobaniec W., Sendrowski K., Olchowik B., Cholewa M., Lukasiewicz A., Lebkowska U. (2011). A volumetric magnetic resonance imaging study of brain structures in children with Down syndrome. Neurol. Neurochir Pol..

[bib59] Teipel S.J., Alexander G.E., Schapiro M.B., Moller H.J., Rapoport S.I., Hampel H. (2004). Age-related cortical grey matter reductions in non-demented Down's syndrome adults determined by MRI with voxel-based morphometry. Brain.

[bib60] Teipel S.J., Schapiro M.B., Alexander G.E., Krasuski J.S., Horwitz B., Hoehne C., Moller H.J., Rapoport S.I., Hampel H. (2003). Relation of corpus callosum and hippocampal size to age in nondemented adults with Down's syndrome. Am. J. Psychiatry.

[bib61] Vandekar S.N., Shinohara R.T., Raznahan A., Roalf D.R., Ross M., Deleo N., Ruparel K., Verma R., Wolf D.H., Gur R.C., Gur R.E., Satterthwaite T.D. (2015). Topologically dissociable patterns of development of the human cerebral cortex. J. Neurosci..

[bib62] Villemagne V.L., Ataka S., Mizuno T., Brooks W.S., Wada Y., Kondo M., Jones G., Watanabe Y., Mulligan R., Nakagawa M., Miki T., Shimada H., O'Keefe G.J., Masters C.L., Mori H., Rowe C.C. (2009). High striatal amyloid beta-peptide deposition across different autosomal Alzheimer disease mutation types. Arch. Neurol..

[bib64] White N.S., Alkire M.T., Haier R.J. (2003). A voxel-based morphometric study of nondemented adults with Down Syndrome. Neuroimage.

[bib65] Wisniewski K.E. (1990). Down syndrome children often have brain with maturation delay, retardation of growth, and cortical dysgenesis. Am. J. Med. Genet. Suppl..

