# Supplementary material for: The Down syndrome brain in the presence and absence of fibrillar β-amyloidosis
Source: Neurobiol Aging. 2017 May;53:11–9. doi: 10.1016/j.neurobiolaging.2017.01.009 (PMC5391869; doi:10.1016/j.neurobiolaging.2017.01.009)
Supplement: Supplementary Table 1 and Supplementary Figs. 1–3 [file mmc1.pdf]

Cross–sectional morphometry studies in Down syndrome

Study	Participants/ demented	Age range (years)	Structural analysis	Regions with lower GM in DS	Regions with increased GM in DS
**Children and adolescents with Down syndrome**					
Pinter *et al.* (2001)	16/0 DS 15 TDC	5–23	ROI analysis using manual volumetry	Whole brain, cerebellum	Parietal lobe, basal ganglia (incl. thalamus)
Menghini *et al.* (2011)	18/0 DS 12 TDC	12–19	VBM using SPM2	Whole brain, L posterior cerebellum, R inferior temporal gyrus, fusiform gyrus, R hippocampus	L anterior cerebellum, R fusiform gyrus, putamen, caudate, insula, superior frontal gyrus, R superior and middle temporal gyrus, inferior frontal gyrus
Smigielska-Kuzia *et al.* (2011)	23/0 DS 26 TDC	2–15	ROI analysis using manual volumetry	Frontal lobe, temporal lobe, hippocampus, amygdala	None found
**Non–demented adults with Down syndrome**					
Weis *et al.* (1991)	7/0 DS 7 TDC	30–45	ROI analysis using manual volumetry	Whole brain, cerebellum	None found
Kesslak *et al.* (1994)	13/0 DS 10 TDC	23–51	ROI analysis using manual volumetry	Frontal cortex, cerebellum, hippocampus,	Parahippocampal gyrus
Raz *et al.* (1995)	13/0 DS 12 TDC	22–50	ROI analysis using manual volumetry	Whole brain, cerebellum, hippocampus	Parahippocampal gyrus
Aylward *et al.* (1997b)	22/0 DS 22 TDC	25–60	ROI analysis using manual volumetry	Whole brain	Putamen
Krasuski *et al.* (2002)	34/0 DS 33 TDC	25–64	ROI analysis using manual volumetry	Amygdala, hippocampus, posterior parahippocampal gyrus	None found
Teipel *et al.* (2003)	34/0 DS 31 TDC	25–64	ROI analysis using manual volumetry	Whole brain, hippocampus	None found
White *et al.* (2003)	19/0 DS 11 TDC	34–56	VBM using SPM99	Whole brain, cerebellum, L medial frontal lobe, R superior/middle temporal lobe, cingulate gyrus, L hippocampus	Some evidence for L parahippocampal gyrus
Beacher *et al.* (2010)	39/0 DS 42 TDC	18–66	ROI analysis using manual volumetry	Whole brain, L frontal lobe, cerebellum	Parietal lobe, putamen, occipital lobe

Supplementary Table 1 continued on reverse

Supplementary Table 1 continued

Study	Participants/ demented	Age range (years)	Structural analysis	Regions with lower GM in DS	Regions with increased GM in DS
**Non–demented and demented adults with Down syndrome**					
Roth *et al.* (1996)	30/10 DS<br>30 TDC	23–60	Visual scoring for atrophy	Basal ganglia	None found
Frangou *et al.* (1997)	17/4 DS<br>17 TDC	30–60	ROI analysis using manual volumetry	Whole brain, planum temporale	None found
Aylward *et al.* (1997a)	30/5 DS<br>30 TDC	25–63	ROI analysis using manual volumetry	Whole brain, cerebellum	None found
Pearlson *et al.* (1998)	50/11 DS<br>23 TDC	Not reported	ROI analysis using manual volumetry	Whole brain, hippocampus, amygdala	None found
Aylward *et al.* (1999)	25/8 DS<br>25 TDC	26–59	ROI analysis using manual volumetry	Hippocampus, amygdala	None found
Prasher *et al.* (2003)	24/11 DS<br>0 TDC	26–78	ROI analysis using manual volumetry	Some evidence for temporal lobe	None found
Beacher *et al.* (2009)	58/19 DS<br>0 TDC	16–66	ROI analysis using manual volumetry	Whole brain, hippocampus, R amygdala, caudate, putamen	None found
Mullins *et al.* (2013)	64/19 DS<br>128 TDC	Not reported	ROI analysis using manual volumetry	Whole brain, hippocampus	None found

Supplementary Table 1 An overview of previously published morphometry studies in adults with Down syndrome (DS). DS – participants with DS; TDC – typically developing control participants; GM – grey matter; L – left; R – right; ROI – region–of–interest; VBM – voxel–based–morphometry; SPM99/2 – Statistical Parametric Mapping neuroimaging analysis software, versions 99 and 2, respectively.

Mean cortical thickness across groups

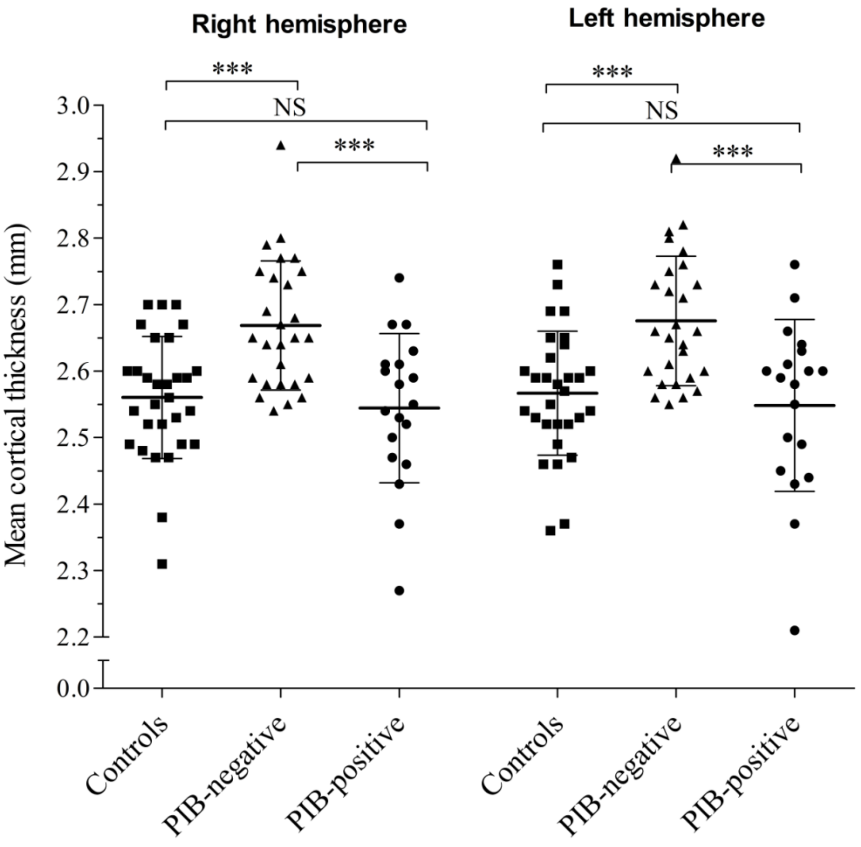

Supplementary Figure 1 The distribution of mean cortical thickness data of the left and right hemispheres in fibrillar β–amyloid negative (PIB–negative) and positive (PIB–positive) individuals with Down syndrome and typically developing controls (Controls). *** – $p<0.001$; NS – non–significant, both Independent sample t–tests (two-tailed).

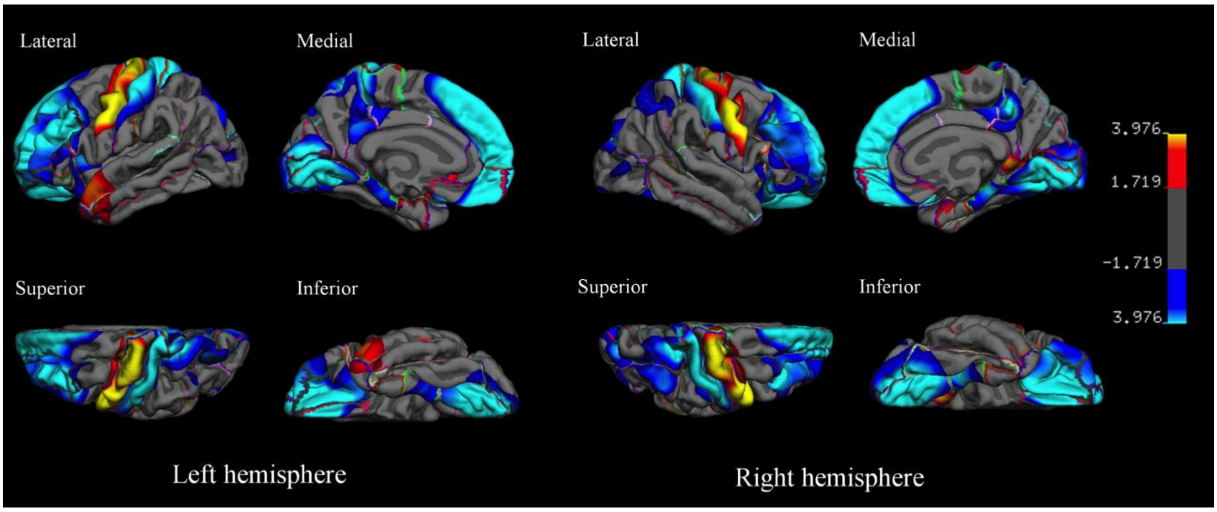

Supplementary Figure 2 The pattern of regional variations in cortical thickness across the hemispheres in the PIB–negative group (n=27) in comparison to control group (n=30), adjusted for differences in age. The colour scale on the right represents the significance of the thickness difference as –log10(p–value) with yellow indicating regions of most significant thinner cortex and light blue indicating regions with most significant thicker cortex in the PIB–negative group compared to Controls. The results are false discovery rate corrected at $p<0.05$ with mean–adjusted age included in the statistical model as a nuisance covariate.

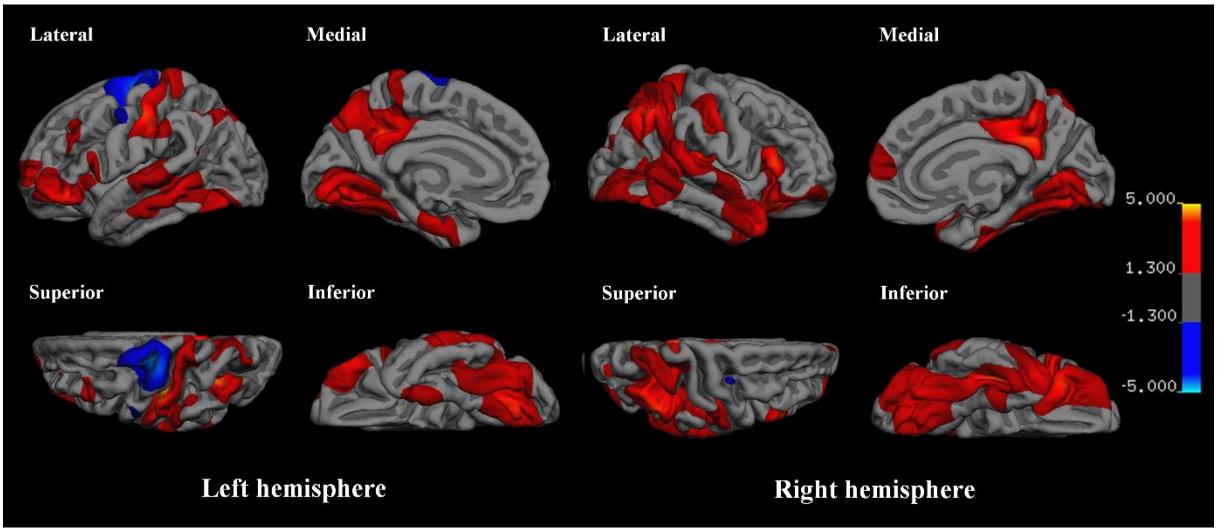

Supplementary Figure 3 The pattern of regional variations in cortical thickness in a sub–group analysis of PIB–negative (n=13) and PIB–positive (n=9) individuals aged between 39 and 48 years, matched for age (Independent sample t–test, t(20)=1.481, p=0.154). The colour scale on the right represents the significance of the thickness difference as –log10(p–value) with yellow indicating regions of most significantly thinner cortex and light blue indicating regions with most significantly thicker cortex in the PIB–positive group compared with PIB–negative group. The results are shown at uncorrected level of $p<0.05$.
