# Supplementary material for: The Down syndrome brain in the presence and absence of fibrillar β-amyloidosis
Source: Neurobiol Aging. 2017 May;53:11–9. doi: 10.1016/j.neurobiolaging.2017.01.009 (PMC5391869; doi:10.1016/j.neurobiolaging.2017.01.009)
Supplement: Supplementary References [file mmc2.doc]

Supplementary References

Aylward, E. H., Habbak, R., Warren, A. C., Pulsider, M.B., Barta, P.E., Jerram, M., Pearlson, G.D., Cerebellar volume in adults with Down syndrome, *Arch**. Neurol**.* 1997a, 54, 209–212.

Aylward, E. H., Li, Q. A., Habbak, R., Warren, A., Pulsifer, M. B., Barta, P. E., Jerram, M., Pearlson, G.D., Basal ganglia volume in adults with Down syndrome, *Psychiatry Res. Neuroimaging* 1997b, 74, 73–82.

Aylward, E. H., Li, Q., Honeycutt, N. A., Warren, A. C., Pulsifer, M. B., Barta, P. E., Chan, M.D., Smith, P.D., Jerram, M., Pearlson, G.D., MRI volumes of the hippocampus and amygdala in adults with Down's syndrome with and without dementia, *Am**. J**. Psychiatry* 1999, 156, 564–568.

Beacher, F., Daly, E., Simmons, A., Prasher, V., Morris, R., Robinson, C., Lovestone, S., Murphy, K., Murphy, D.G.M., Alzheimer's disease and Down's syndrome: an in vivo MRI study, *Psychol**. Med**.* 2009, 39, 675–684.

Beacher, F., Daly, E., Simmons, A., Prasher, V., Morris, R., Robinson, C., Lovestone, S., Murphy, K., Murphy, D.G.M., Brain anatomy and ageing in non-demented adults with Down's syndrome: an in vivo MRI study, *Psychol**. Med**.* 2010, 40, 611–619.

Frangou, S., Aylward, E., Warren, A., Sharma, T., Barta, P., Pearlson, G., Small planum temporale volume in Down's syndrome: a volumetric MRI study, *Am**. J**. Psychiatry* 1997, 154, 1424–1429.

Kesslak, J. P., Nagata, S. F., Lott, I., Nalcioglu, O., Magnetic resonance imaging analysis of age-related changes in the brains of individuals with Down's syndrome, *Neurology* 1994, 44, 1039–1045.

Krasuski, J. S., Alexander, G. E., Horwitz, B., Rapoport, S. I., Schapiro, M. B., Relation of medial temporal lobe volumes to age and memory function in nondemented adults with Down's syndrome: implications for the prodromal phase of Alzheimer's disease, *Am**. J**. Psychiatry* 2002, 159, 74–81.

Menghini, D., Costanzo, F., Vicari, S., Relationship between brain and cognitive processes in Down syndrome, *Behav**. Genet**.* 2011, 41, 381–393.

Mullins, D., Daly, E., Simmons, A., Beacher, F., Foy, C., Lovestone, S., Hallahan, B., Murphy, K.C., Murphy, D.G., Dementia in Down’s syndrome: an MRI comparison with Alzheimer’s disease in the general population, *J**. Neurodev. Disord**.* 2013, 5, 19.

Pearlson, G. D., Breiter, S. N., Aylward, E. H., Warren, A. C., Grygorcewicz, M., Frangou, S., Barta, P.E., Pulsifer, M.B., MRI brain changes in subjects with Down syndrome with and without dementia, *Dev**. Med**.* *Child Neurol**.* 1998, 40, 326–334.

Pinter, J. D., Eliez, S., Schmitt, J. E., Capone, G. T., Reiss, A. L., Neuroanatomy of Down's syndrome: a high-resolution MRI study, *Am**. J**. Psychiatry* 2001, 158, 1659–1665.

Prasher, V., Cumella, S., Natarajan, K., Rolfe, E., Shah, S., Haque, M. S., Magnetic resonance imaging, Down's syndrome and Alzheimer's disease: research and clinical implications, *JIDR* 2003, 47, 90–100.

Raz, N., Torres, I. J., Briggs, S. D., Spencer, W. D., Thornton, A. E., Loken, W. J., Gunning, F.M., McQuain, J.D., Driesen, N.R., Acker, J.D., Selective neuroanatomic abnormalities in Down's syndrome and their cognitive correlates: evidence from MRI morphometry, *Neurology* 1995, 45, 356–366.

Roth, G. M., Sun, B., Greensite, F. S., Lott, I. T., Dietrich, R. B., Premature aging in persons with Down syndrome: MR findings, *Am**. J**. Neuroradiol.* 1996, 17, 1283–1289.

Smigielska-Kuzia, J., Bockowski, L., Sobaniec, W., Sendrowski, K., Olchowik, B., Cholewa, M., Lukasiewicz, A., Lebkowska, U., A volumetric magnetic resonance imaging study of brain structures in children with Down syndrome, *Neurol**. Neurochir Pol**.* 2011, 45, 363–369.

Teipel, S. J., Schapiro, M. B., Alexander, G. E., Krasuski, J. S., Horwitz, B., Hoehne, C., Moller, H.J., Rapoport, S.I., Hampel, H., Relation of corpus callosum and hippocampal size to age in nondemented adults with Down's syndrome, *Am**. J**. Psychiatry* 2003, 160, 1870–1878.

Weis, S., Weber, G., Neuhold, A., Rett, A., Down syndrome: MR quantification of brain structures and comparison with normal control subjects, *Am**. J**. Neuroradiol.* 1991, 12, 1207–1211.

White, N. S., Alkire, M. T., Haier, R. J., A voxel-based morphometric study of nondemented adults with Down Syndrome, *Neuroimage* 2003, 20, 393–403.
